# The Integration of Veterinary Medicine and Behavioral Management in the Care of Captive Pigtail Macaques (*Macaca nemestrina*)

**DOI:** 10.3390/vetsci11100465

**Published:** 2024-10-01

**Authors:** Jessica E. Toscano, Sarah A. Hart, Carolyn M. Malinowski

**Affiliations:** 1 Arizona Breeding Center, Washington National Primate Research Center, University of Washington, Mesa, AZ 85215, USA; jesst393@uw.edu (J.E.T.); sarah105@uw.edu (S.A.H.); 2 Michale E. Keeling Center for Comparative Medicine and Research, The University of Texas MD Anderson Cancer Center, Bastrop, TX 78602, USA

**Keywords:** pigtail macaque, nonhuman primate, behavior, positive reinforcement training

## Abstract

**Simple Summary:**

The use of animals in biomedical research for the development of treatments, drugs, medical devices, and other therapies is required by the government though the Federal Food, Drug, and Cosmetic Act. While one of the less-commonly utilized species, nonhuman primates play a vital role in this process as they are more closely related to humans anatomically and genetically than other animals. Because nonhuman primates are more complex and have longer life spans than other species, it is crucial to address their psychological and social needs. The use of positive reinforcement, social housing, and enrichment strategies are some of the most efficacious methods commonly implemented to address nonhuman primate psychological well-being. Here, we describe these methodologies and how they offer animals more control and choice in their movements, affects, and participation in daily activities, thereby resulting in decreased stress and anxiety.

**Abstract:**

The Washington National Primate Research Center (WaNPRC) maintains the largest domestic breeding colony of pigtail macaques (*Macaca nemestrina*) in the United States, with animals housed in small to medium-sized social groups. As part of the animal care plan, a programmatic framework is utilized, which integrates clinical care with socialization considerations for nonhuman primates (NHPs). This framework encompasses the following areas: (1) socialization in the clinical setting; (2) positive reinforcement training (PRT); (3) measures to ensure proper identification and medication distribution; and (4) in-group treatments. The success of this framework is demonstrated by the high socialization rate for hospitalized animals (99.5% social pairing success), with the majority of clinical cases (95%) being treated in social groups. Ultimately, this framework seeks to buffer stressors when animals require clinical care or husbandry manipulations. Taken together, the above components foster an environment that provides a comprehensive approach to NHP medical and behavioral management.

## 1. Introduction

The Washington National Primate Research Center (WaNPRC) is one of seven federally funded National Primate Research Centers dedicated to identifying causes, preventative measures, treatment options, and cures for diseases and conditions that impact millions of lives around the world [1]. WaNPRC exists as a research unit within the Office of Research at the University of Washington (UW) and performs life-saving research in the areas of neuroscience, infectious disease and translational medicine, gene therapy, and regenerative medicine, as well as global conservation, education, and outreach [2]. To achieve WaNPRC’s mission of empowering leading-edge research, a breeding colony of pigtail macaques (*Macaca nemestrina*) is maintained to provide healthy animals for research to investigate, understand, treat, and cure diseases and conditions that affect both humans and animals. The majority of WaNPRC’s breeding animals are housed at an off-site location in Mesa, Arizona, which, because of the climate, allows animals to be housed in outdoor enclosures all year.

The Arizona Breeding Center maintains a colony of approximately 450 animals housed in small to medium-sized social groups in either indoor/outdoor or indoor-only environments. Of these animals, 162 breeding-aged females are housed in harem groups with group sizes ranging from 5 to 16 animals. At present, there are 20 harem breeding groups at WaNPRC. The rest of the colony is composed of dam–infant groups with no adult breeder male present, or juvenile groups of animals. All animals receive daily care by trained husbandry, behavioral, and veterinary staff and are housed according to the regulations and standards outlined by the USDA Animal Welfare Act and Regulations [3,4] and the Guide for the Care and Use of Laboratory Animals [5]. The UW Institutional Animal Care and Use Committee (IACUC) and the Attending Veterinarian oversee animal care and approve procedures. Additionally, the UW is an AAALAC-Accredited Program.

It is well established and understood that social housing provides the best form of enrichment for nonhuman primates (NHPs) [5,6,7,8]. Social stimulation enables the activation of all sensory systems and provides the animals with the greatest opportunity to express normal species-specific social interactions and behavioral repertoires [9,10]. Social deprivation, even for a short amount of time, results in physiological and clinical changes in NHPs, including self-directed aggression, immunological changes, increased blood pressure, depression, and increased anxiety [11,12,13,14,15,16,17]. Additionally, all regulatory agencies in the United States endorse social housing for NHPs utilized in research [18]. While there is a regulatory provision for justified exemptions of NHPs from social housing either by the IACUC to meet the needs of research protocols or by the Attending Veterinarian for animal clinical conditions or animal well-being, WaNPRC only employs this provision when absolutely necessary as deemed by a veterinarian experienced in NHP medicine and care or if the investigator has an IACUC-approved justified exemption [3]. In all other circumstances, every effort is taken to either keep animals in groups or provide socialization when in the hospital for clinical care.

Positive reinforcement training (PRT) is a well-recognized refinement to laboratory animal welfare that is used as a tool to facilitate interactions between care staff and animals [19,20,21,22,23]. PRT relies on the voluntary participation of the animal with the specific aim of creating a cooperative environment that may otherwise be a source of stress, such as simple medical procedures to monitor physical health or animal movements for husbandry needs. Cooperative care via PRT is a beneficial refinement as it improves research results by reducing possible stress confounds and desensitizes animals to potentially stressful events [20,24,25]. Reducing the number of routine clinical procedures that require a sedation event would therefore reduce components of psychological and physiologic stress associated with momentary restraint and sedatives, such as pain on injection and volume-dependent tissue damage [26]. Cooperative care also reduces the need to remove animals from their social groups, ultimately minimizing social disruption for multiple animals.

WaNPRC believes in and supports the integration of and collaboration between veterinary medicine and behavioral management toward the goal of providing the best possible environments and care for the animals to ensure excellent scientific outcomes. At WaNPRC, the behavioral management and veterinary teams work closely together both in the oversight and provision of care for animals in groups and those in single or pair housing while under care in hospital settings. This teamwork encompasses the following areas: (1) socialization in the clinical setting; (2) positive reinforcement training to encourage voluntary participation during animal movements, husbandry activities, and clinical procedures; (3) measures to ensure proper animal identification and medication distribution; and (4) in-group treatments. This framework seeks to buffer stressors when animals require clinical care or husbandry manipulations. Here, the programmatic framework is described for treating animals in their social groups, socialization while in the hospital, and our positive reinforcement training program. These components foster an environment that provides a comprehensive approach to NHP medical and behavioral management at WaNPRC.

## 2. Materials and Methods

### 2.1. Socialization in the Clinical Setting

**Figure A1:**
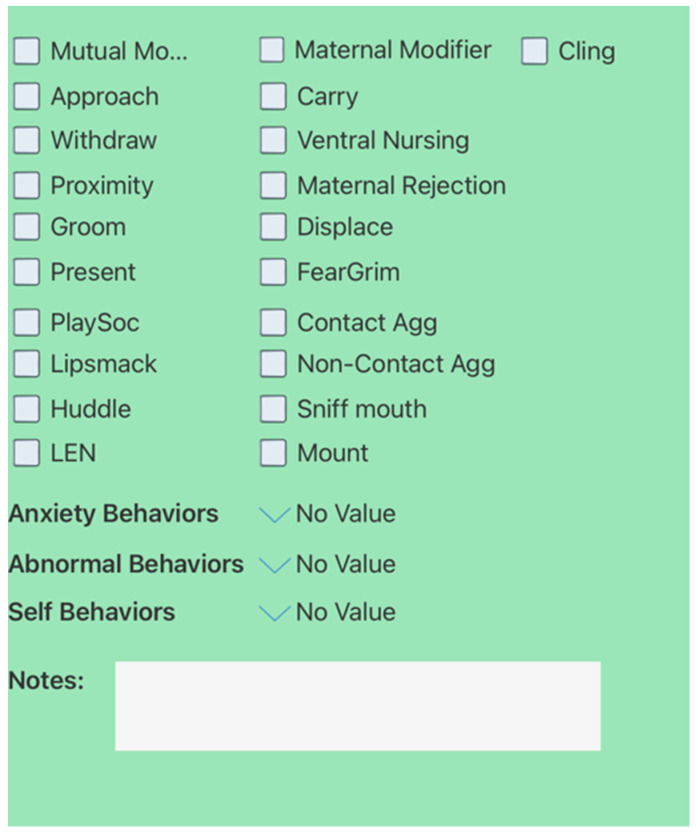
HanDBase pair data collection module. The module utilized to collect behavioral observations and data for animals socialized in the hospital.

To appreciate the benefits of socialization while in the hospital, a data collection strategy was designed to document behaviors for animals pair-housed in the hospital via continuous scan sampling for 15 min [27]. Behavioral data were collected by two trained observers, demonstrating an interobserver reliability of 90% over 30 observations ([agreements/[agreements/disagreements]) × 100). A hospital socialization ethogram consisting of 30+ behaviors and aimed to capture a wide array of behaviors was utilized during scan sampling (Table A1). A three-tiered data collection paradigm was implemented in which each pair was observed for 15 min: 3 times via in-person observations and 3 times via a remote live feed for a total of 6 observations per pair. Observations commenced the first day the pair was established, 1–3 days after the pair was established, and approximately one week after the pair was established. All data were collected using HanDBase (DDH Software, LLC, Atlanta, GA, USA) (Figure A1), which allowed for the identity of the initiator and recipient of a social interaction to be recorded, along with self-directed behaviors at the individual level. Collecting data in this manner enabled further assessment of any differences between animals paired with familiar partners (i.e., animals from the same social cohort) and those that were paired with novel partners (i.e., animals not from the same social cohort), and whether the clinical reason for the relocation impacted the formation, or outcome, of the pairing. That is, the reason for the hospitalization (trauma vs. gastrointestinal issues) may impact the rate of behaviors observed in the pairing, which is an area of exploration currently underway. Criteria of a successful pairing were arbitrarily defined as developing a clear rank relationship, no observable retreating behavior, no trauma, observed affiliation, and remaining paired for the duration of hospital stay. It is important to note that these hospital socializations were transient, and the length of time paired was variable, though none of the pairs failed while in the hospital. It would be of interest to assess if the length of time together influenced any behavioral patterns exhibited by the hospital pairs. Once an animal was released from hospital care, they returned to their social group. If the social partner remained in the hospital, it was available to be paired with another animal.

### 2.2. Positive Reinforcement Training Program

The behavior and veterinary care teams worked together to collaboratively identify areas of care that would benefit from positive reinforcement training, and a shaping plan for the desired behavior was created. These shaping plans detail what the final behavior will look like, as well as the broken-down approximations that will result in the desired final behavior. Breaking the behavior down into smaller steps sets the animal up for success by maintaining high reward rates [28]. The behavior-shaping plans detail what reinforcement will be used, typically a food item preferred by the individual, including, but not limited to, small produce items, Gatorade, or gummy bears. The frequency of sessions was noted, as well as potential solutions when working with more timid or aggressive animals. Additionally, desensitization and/or time to habituation was assessed at each approximation. Habituation is defined as how long animals coordinated a specific phenotypic response of adaptation to new stimuli.

At WaNPRC, collaborative training opportunities utilizing PRT and cooperative care were opportunistically developed between behavioral management and veterinary services. One such opportunity presented itself when the need arose to section adult males from their groups to facilitate clinical and husbandry activities. Sectioning a male involves targeting him to a desired location within the enclosure and closing a door while he remains stationed in said desired location, essentially separating him from the rest of the enclosure and his group mates. The shift-training shaping plan includes approximations of target training, where the animal touches and moves with a target pole and stations at a desired location while a manual shift door is closed. Stationing for door closure was broken down into smaller approximations, which included reinforcing for stationing while the keys were manipulated, the shift door was unlocked, and during partial door closure, all of which worked up to the desired behavior of full door closure.

Shifting and stationing plans were also utilized to provide prescribed medications to animals while they remained in their social groupings. Veterinary staff were trained by the behavior team until deemed proficient by a member of BMS to use verbal or visual cues to encourage animals to relocate themselves to a certain location in the enclosure to distract them when lower-ranking animals were receiving clinical items. This technique is often employed with higher-ranking animals to encourage them to stay in one location to be stationed or temporarily sectioned off to ensure lower-ranking animals can receive their treatment. Groups are often manipulated such that lower-ranking animals are sectioned off within their enclosure to facilitate medication distribution while providing them a secure space to consume their treatment without fear of aggression or intimidation from higher-ranking animals during consumption. This also prevents the normal behavior of lower-ranking animals giving their treatments to higher-ranking animals.

The following case study highlights the behavioral management and veterinary interface. A low-ranking female was reluctant to take her treatment in the presence of higher-ranking animals. The behavior team trained the animal to accept her treatment in the outdoor portion of the enclosure while stationed on a rope and away from her group. This method was repeated twice daily by behavior staff until she would accept and consume her treatment consistently. The medication distribution was then transferred to veterinary staff to assess if the animal would generalize acceptance of her medications regardless of the person distributing them. This case study highlights that the behavior team was able to serve as a bridge to assist with animal treatment compliance as needed. Once deemed proficient, veterinary staff were re-incorporated in the process and resumed medication distribution. Throughout the entire process, the behavior and veterinary teams were in regular communication with what worked best for the individual animal, with the ultimate goal being the reliable consumption of medications by the animal, thereby minimizing the need for clinical relocation.

Another collaborative opportunity presented itself in 2023 when four breeder females were selected by a veterinarian and trained to present for awake cage-side ultrasounds, with the goal to closely monitor pregnancies without sedatives or repeatedly removing the females from their social group. The selected animals had no prior training history. To establish the behavior, the animals were first desensitized to the trainer by positive reinforcement for approaching. Approximations in the ultrasound-training shaping plan included the shifting behavior, targeting and stationing the animal into an area of the enclosure and subsequently sectioning them off from the rest of the social group with a manual shift door while remaining in visual and mesh access with their group. To achieve awake abdominal ultrasounds, the following approximations were implemented: targeting to the mesh, stationing for abdominal touch with the verbal cue “touch” using a target pole, abdominal touch with ultrasound gel added to the target pole to habituate the animals to the texture and temperature of the gel, stationing for abdominal touch with the ultrasound probe and adding ultrasound gel to the probe, and finally stationing for a cage-side abdominal ultrasound performed by behavioral management services. When scans could be captured, they were then reviewed by the veterinarian. The final approximation in this trained behavior was bridging the behavior to the veterinarian.

## 3. Results

### 3.1. Socialization in the Clinical Setting

**Figure 1:**
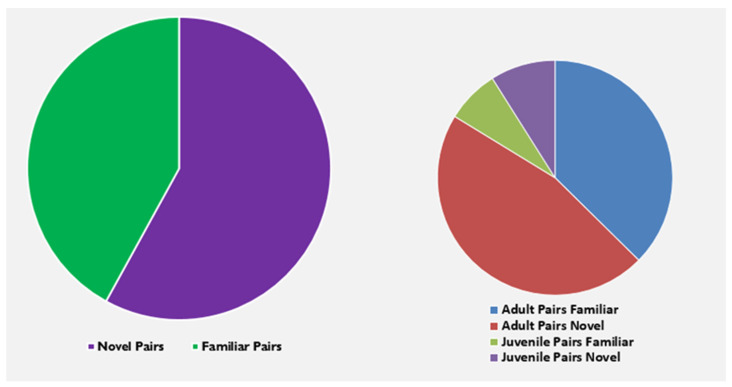
The composition of hospital pairs from 2021 to 2022 demonstrates the breakdown of pairs that were novel to each other versus those that had a pre-existing social history, along with a further delineation of age class by familiarity.

Prior to 2020, animals were not regularly socialized while in the hospital. Beginning in 2021, a standard hospital socialization program was implemented to address this lack of socialization. Between 2020 and 2021, the number of hospitalized socializations increased from 19 pairs in 2020 to 123 pairs in 2021. In 2022, the same success rate was maintained, with 123 pairs established. Of the 246 pairs established in 2021 and 2022, a success rate of 99.5% was achieved. It is important to note that the total number of pairs established was counted when a unique combination of animals was socialized. The 246 pairs do not represent 492 individual animals, nor do they represent individual hospital clinical cases. That is, the same animal may have been paired several times in the hospital, but with a different animal each time, depending on the length of the hospital stay and required clinical care. Additionally, not all animals were in the hospital for clinical care. Hospital caging was also utilized for housing animals awaiting new social groups as well as for animals being prepared for shipment. Overall, 42% of pairs were familiar and from the same group, while 58% were novel pairings. The mean duration of socialization while in the hospital was 23 days ($\bar{x}$ = 23.88, ±27.29 days). Animals were deemed socially compatible by the behavior team typically 1–2 days after being introduced, which represents the first day of the start of the pairing. A rapid pairing strategy was implemented in which animals were often in protected contact (i.e., wide mesh grate for approximately 15 min then opened up to full-access). The age-class composition of the 246 pairs established within the specified timeline was as follows: 187 of the 246 pairs established were adult–adult pairs, with the majority being female–female pairs; 54 of the 246 pairs established were juvenile–juvenile pairs; and 5 of the 246 pairs were adult–juvenile pairs (Figure 1). A chi-square test of independence was performed to examine the relation between familiarity and age class for pairs established in 2022. There were no statistical differences in the success of pairings based on the familiarity and age class of the partnership. The relation between these variables was not significant, *X*^2^ (1, *N* = 123) = 0.0008, *p* = 0.9777.

### 3.2. Positive Reinforcement Training Program

Of the 20 males currently in harem breeding, 13 became proficient at shifting cooperatively. The mean total training time to reach proficiency for the 13 breeder males was 87 min ($\bar{x}$ = 87.3 ± 91.1 min) in eight sessions ($\bar{x}$ = 8 ± 5.7 sessions). Individual training sessions lasted 9.6 min ($\bar{x}$ = 9.6 ± 2.8 min) and took place 4–5 times a week depending on animal cooperation. Proficiency was defined as the animal successfully cooperating in the desired behavior over three consecutive training sessions. The remaining seven males currently have shift training cases in progress.

At present, approximately 95% of clinical cases are successfully managed in groups. The veterinary team performs the majority of the in-group treatment distributions. In rare cases, the behavior team may administer the medications if the animal more readily takes medications when distributed by behavior staff. Typically, the behavior team will then assume responsibility for medication distribution for those animals until they demonstrate increased comfort with taking their in-group medications.

**Figure 2:**
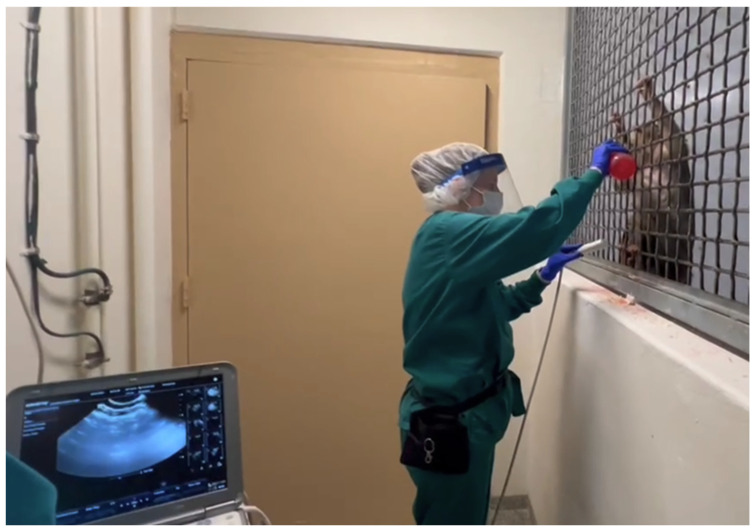
A technician demonstrates using positive reinforcement with a red juice reward to condition an animal to present its abdomen for voluntary abdominal ultrasound scans for pregnancy diagnosis and monitoring.

**Figure 3:**
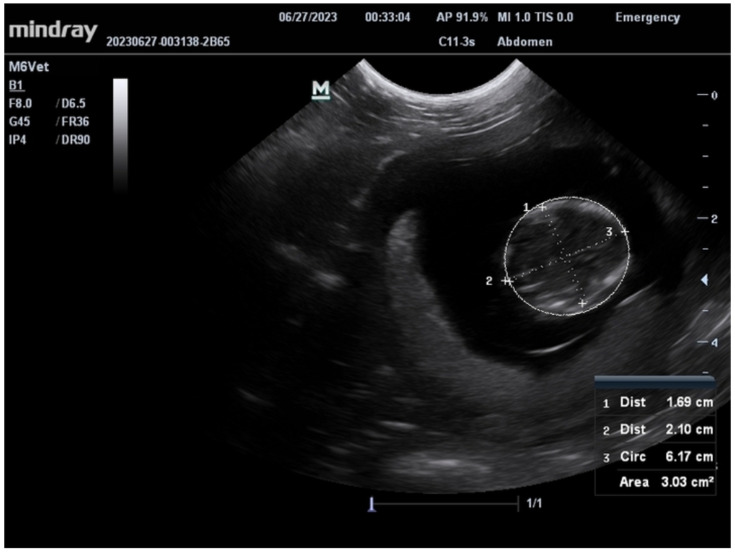
An ultrasound still image demonstrating the bi-parietal (skull) measurement that is used to determine the gestational age of the fetus.

**Figure 4:**
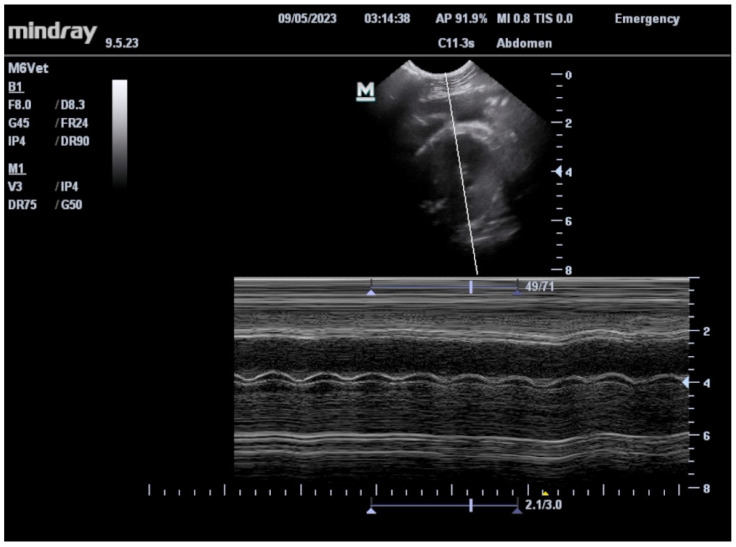
An ultrasound still image demonstrating the measurement of fetal heart rates and contractility that are utilized to determine fetal viability.

All four females voluntarily cooperated in cage-side ultrasounds to assess pregnancy status in approximately 3.5 months with a mean training time of $\bar{x}$ = 441.25 ± 51.2 min or $\bar{x}$ = 40.75 ± 4.5 sessions (Figure 2). The mean duration of each training session was 11.25 min ($\bar{x}$ = 11.25 ± 1.26 min), with sessions scheduled 3–4 times a week with variation in animal voluntary cooperation. Once confirmed pregnant, subsequent scans enabled the determination of the gestational due date via bi-parietal diameter measurements (Figure 3), measurement of fetal heart rates (Figure 4), assessment of placenta and amniotic fluid, identification of fetal movement, and additional fetal skeletal measurements. This behavior was successfully bridged to our veterinarians by three of the four females with a mean number of training sessions of $\bar{x}$ = 5 ± 2.65 after proficiency was first achieved by the behavior team. The fourth female gave birth prior to proficiency being reached with the veterinarian. The estimated due dates of the other three females obtained by awake cage-side ultrasound scans were within 18 days of their actual births. This is comparable to the 8 days of accuracy found for sedated ultrasound scans for a random sample of three females of the same age range (8–9 years old) from the colony. Estimated due dates are typically scheduled for reassessment at +21 days, so this range is within the normal limits.

## 4. Discussion

The veterinary care of captive pigtail macaques in this setting revolves around the following major categories: (1) diarrhea and weight loss; (2) trauma; (3) endemic coccidioidomycosis (Valley Fever); and (4) arthritis management for geriatric animals. At any given time, approximately 10% of the colony animals are under treatment for one or more of the above conditions. Every effort is made to treat the animal in situ in its social group, unless the condition requires veterinary management in a hospital setting. A holistic approach is utilized for each case to consider both the veterinary and behavioral needs of the animal, with socialization being the norm for hospitalized cases. In cases involving high-ranking animals, particular attention is given to minimize any stability issues due to removing a socially important animal from the group, often with treatment modifications to minimize time away from the group.

A highlight of the veterinary program is treating animals in their social groups. The primary goal is to minimize stress that may be associated with relocating animals out of their home enclosures, therefore maximizing animal compliance with treatments in their social groups under veterinary discretion and reducing stress that could compromise the effectiveness of clinical care. Most clinical cases are managed with daily oral medications that can be administered to animals in the group setting. To ensure the correct animals receive their prescribed treatments, veterinary staff must demonstrate the ability to identify 30 animals randomly selected by the behavior team and must exhibit knowledge of social rank and group dynamics, both of which are imperative to distribute in-group treatments effectively. To minimize drift, this identification assay is completed annually by veterinary staff. To encourage compliance in consuming treatments, technicians use creative methods to make vehicles for medication administration to mask bitter tastes, take note of individual animal preferences, and modify ingredients daily as needed. Veterinary and behavioral staff work closely together on individual cases to ensure each animal receives its prescribed medications. Overall, a collaborative and communicative effort and a detailed working knowledge of group dynamics facilitates the ability to achieve a high success rate of in-group treatments at WaNPRC.

When animals are unable to be treated in-group due to presenting medical conditions, they are relocated to a hospital room to receive veterinary care. While in the hospital, animals are regularly socialized to buffer stress associated with veterinary interventions and relocations. There are many documented findings which show that consistently housing NHPs with social contact has a wide scope of benefits, ranging from health outcomes to fostering the ability to express species-typical behaviors [6,9,10,29,30]. Additionally, social contact during periods of stress reduces both anxiety-related and abnormal behaviors [11,14,15]. The principle of socialization is adhered to, even while temporarily in the hospital, to buffer stressors associated with being in a novel environment when removed from their respective social groups. Social buffering results in reduced stress and potentially improved appetite, activity, treatment compliance, and reduced time in hospital, as well as improved clinical and behavioral outcomes. Furthermore, socialization does not hinder clinical case management. The caging utilized allows for animals to be temporarily separated to determine individual fecal production, to sedate animals for treatments, or to ensure medication consumption. All hospital socializations are approved by a veterinarian experienced in primate medicine. The general framework for socialization eligibility is as follows: animals removed from their respective groups for non-clinical reasons, such as holding for upcoming group introductions, can be immediately socialized; animals removed with trauma are eligible for pairing shortly after admission; animals with unspecified weight loss are eligible for pairing; and animals with diarrhea/loose stools are not immediately eligible on admission to the hospital due to possible contagion, unless the animals are from the same group or if the etiology of the diarrhea is suspected to be similar between cases and animals are at the same point in their course of treatment. The high success rate of the hospital pairing program is being further explored by assessing the quality of these pairings via a three-phase data collection paradigm. Further theoretical avenues of exploration include examining if time spent in group housing may result in animals being more amenable to dyadic socialization (i.e., they are motivated to socialize with conspecifics) and determining if there is an interaction between species and success of pairings that is unique to pigtail macaques.

It is important to note that previous behavioral data taken on animals when in hospital were opportunistic and utilized one-zero sampling [27]. When one-zero sampling is used to record behaviors, it does not matter whether the behavior occurred once or several times during the time interval. This method is somewhat limited in its usefulness because information is lost by categorizing the occurrence of behaviors so rigidly. Applying all-occurrence sampling is more useful in determining the rate, frequency, or synchrony of the occurrence of specific behaviors [27]. Future plans include collecting data on singly housed hospitalized animals using this same methodology to parse out differences in the overall frequency of abnormal and anxiety-related behaviors when socialized versus singly housed.

As previously documented in other NHP species, incorporating PRT into the daily routine is vital at WaNPRC when animal movements are necessary and has proven beneficial for both husbandry and clinical needs [19,20,22,23,24,25,28,31,32,33,34,35,36]. Utilizing the shifting and sectioning of adult breeder males allows for animal care staff to safely enter the enclosure to shift the remaining animals for cleaning and enables veterinary services to distribute treatments to members of the group without potential interference from the male, as well as being a component of a larger trained behavior that involves breeder males cooperatively transferring from their home enclosure into portable caging. Having this trained behavior in their repertoire has been valuable whenever the group requires manipulation, particularly when the transfer behavior in breeder males is utilized the day prior for routine semi-annual health exams. Shifting males to a separate enclosure the day prior to exams allows for males to be positively reinforced with preferred food rewards, which would not be possible the day of the exam due to health protocols that require animals to be fasted the morning of exams.

While it is well documented in many NHP species, there is little information available on pigtail macaques regarding socialization and the use of PRT [6,9,10,11,12,13,14,15,16,19,20,24,25,28,29,30,31,32,33,34]. The future of WaNPRC’s training program aims to continue developing collaborative shaping plans for routine clinical procedures that could be performed with animals remaining in their social groups without the need to sedate, such as body presentation for obtaining swab samples or cardiopulmonary auscultation. WaNPRC is also in the beginning stages of developing cooperative measures to obtain group-housed juvenile and nursery-reared animal body weights by utilizing PRT to encourage animals to voluntarily enter weight boxes to reduce unnecessary animal handling.

## 5. Conclusions

By promoting collaboration and integrating the veterinary care and behavioral management of pigtail macaques, WaNPRC is able to concurrently address the social and behavioral needs of the animals while providing high-quality veterinary care. The Center believes that this comprehensive cooperative approach provides a refinement in the care for its animals by reducing social stressors associated with hospital care and increasing consumption compliance for prescribed medications. This refinement is demonstrated by the high rates of success for in-group treatments, hospital socialization, the cooperative shifting of breeder males, and cage-side ultrasounds in females. Further refinements will focus on collecting quantifiable data on animals socialized in the hospital versus those that are not. Additionally, WaNPRC will continue to implement operant conditioning training to encourage animals to cooperate in medical procedures that can be performed without the use of sedatives or removal from their respective social groups.

## Data Availability

The raw data supporting the conclusions of this article will be made available by the authors on request.

## Appendix A

**Table A1:** Hospital Pair Ethogram. The socialization ethogram utilized to assess and collect data for pair-housed hospitalized animals consists of 30+ behaviors aimed to capture a wide array of behaviors. A three-tiered data collection paradigm was implemented in which each pair was observed for 15 min, 3 times via in-person observations and 3 times via a remote live feed for a total of 6 observations per pair.

Pair Ethogram	
**Social Behaviors**	
Approach	If a monkey comes within an arm’s reach of another.
Carry	Holding an infant while in ventral/ventral or dorsal/ventral contact.
Ventral Contact/Nursing	Infant on dam’s ventrum and visibly suckling.
Cling	Full-body contact with partner(s) and wrapping arms/legs around partner(s), often excessively (one member or more members resist separation).
Groom Other	Picking, scraping, spreading, mouth picking, or licking of a monkey’s hair or skin by another monkey.
Len	A facial display of lips forward, ears back, and neck extended.
Huddle	Calmly sitting in contact with one or more animals. May include sleeping.
Lip Smack	Rapid movement of the lips either up or down or pursing them.
Mount	A monkey grabs the hind legs of another monkey with his/her own hind feet and places his or her hands on the lower back of the recipient. This may also include awkward mounts of other body parts.
Present	Postures used to solicit a behavior from another monkey. Monkeys expose the rump, neck, ventrum, back, or other surface of the body in an exaggerated way. Three types of present are scored: Other, Rump, and Sex.
Proximity	Sitting or standing within an arm’s reach of the other monkey for at least 5 s.
Sniff/Mouth	Sniff, mouth, or olfactory investigation of another monkey’s muzzle.
Social Play	Non-aggressive chasing, bouncing, grabbing, wrestling, soliciting, and mock biting of another monkey. Often seen with an open-mouth “play face”.
Contact Aggression	Hitting, grabbing, scratching, hair-pulling, or biting of another monkey.
Non-Contact Aggression	Head bob, lunge, chase, open mouth, brow flash, ear flap, huff, or staring.
Displace	Taking over another monkey’s position.
Fear Grimace	Lips are pulled back in an exaggerated manner, exposing teeth.
Scream	Loud, shrill vocalization.
Withdraw	Subject monkey walks away due to movement toward it by another monkey.
**Self Behaviors**	
Forage	Picking at floor w or w/o substrate or manipulating edible enrichment.
Groom Self	Auto-groom: Picking at or systematically parting fur with hands, fingers, teeth, or lips.
Manipulate Enrichment	Closely inspects or manipulates non-edible enrichment object (barrel, swing, rope, and toy).
Self-Play	Solitary play (can be with object/s or include locomotion).
**Anxious Behaviors**	
Whole Body Shake	Shaking whole body like a wet dog.
Yawn	Open-mouth yawn; eyes may be closed or animal may not make eye contact with anyone.
Self-Scratch	Scratch with feet or hands; includes rough scratch.
Tooth Grind	Chomping (opening and closing of mouth that makes an audible sound) or tooth grinding (may occur without opening and closing of mouth).
**Abnormal Behaviors**	
Over-Grooming	Over-grooming: hair pulling or plucking with hands, feet, or mouth; may include eating hair.
Stereotypy Non-locomotor	Head tossing; eye covering, poking, or pressing; saluting.
Stereotypy Locomotor	Pacing, flipping, and twirling; bouncing; rocking. At least three consecutive repetitions required.
Stereotypy Locomotor, of Concern	Animal spends approximately or more than half of the observation engaging in LS or LS that is of particular concern to the observer.
Self-Directed Behaviors	Behaviors including self-clasping; self-mouthing; self-sucking; self-sucking or repetitive licking of toes, fingers, or nipples.

## References

[1] National Primate Research Centers. https://nprc.org.

[2] Washington National Primate Research Center. https://wanprc.uw.edu/.

[3] (2008). Animal Welfare Act as Amended. https://uscode.house.gov/view.xhtml?path=/prelim@title7/chapter54&edition=prelim.

[4] (2008). Animal Welfare Regulations. https://www.ecfr.gov/current/title-9/chapter-I/subchapter-A.

[5] Institute for Laboratory Animal Research (2022). Guide for the Care and Use of Laboratory Animals.

[6] Lutz C.K., Novak M.A. (2005). Environmental enrichment for nonhuman primates: Theory and application. ILAR J..

[7] Bernstein I.S. (1991). Social housing of monkeys and apes: Group formations. Lab. Anim. Sci..

[8] Hannibal D.L., Bliss-Moreau E., Vandeleest J., McCowan B., Capitanio J. (2017). Laboratory rhesus macaque social housing and social changes: Implications for research. Am. J. Primatol..

[9] Novak M.A., Suomi S.J. (1991). Social interaction in nonhuman primates: An underlying theme for primate research. Lab. Anim. Sci..

[10] Schapiro S.J., Bloomsmith M.A., Suarez S.A., Porter L.M. (1996). Effects of social and inanimate enrichment on the behavior of yearling rhesus monkeys. Am. J. Primatol..

[11] Novak M.A., Kinsey J.H., Jorgensen M.J., Hazen T.J. (1998). Effects of puzzle feeders on pathological behavior in individually housed rhesus monkeys. Am. J. Primatol..

[12] Coelho A.M., Carey K.D., Shade R.E. (1991). Assessing the effects of social environment on blood pressure and heart rates of baboons. Am. J. Primatol..

[13] Lilly A.A., Mehlman P.T., Higley J.D. (1999). Trait-like immunological and hematological measures in female rhesus across varied environmental conditions. Am. J. Primatol..

[14] Schapiro S.J., Bushong D. (1994). Effects of enrichment on veterinary treatment of laboratory rhesus macaques (*Macaca mulatta*). Anim. Welf..

[15] Schapiro S.J. (2002). Effects of social manipulations and environmental enrichment on behavior and cell-mediated immune responses in rhesus macaques. Pharmacol. Biochem. Behav..

[16] Gonzalez C.A., Coe C.L., Levine S. (1982). Cortisol responses under different housing conditions in female squirrel monkeys. Psychoneuroendocrinology.

[17] Gottlieb D.H., Capitanio J.P., McCowan B. (2013). Risk factors for stereotypic behavior and self-biting in rhesus macaques (*Macaca mulatta*): Animal’s history, current environment, and personality. Am. J. Primatol..

[18] OLAW. https://olaw.nih.gov/faqs#/guidance/faqs?anchor=51331.

[19] Schapiro S.J., Magden E.R., Reamer L.A., Mareno M.C., Lambeth S.P., Weichbrod R.H., Thompson G.A.H., Norton J.N. (2018). Behavioral Training as Part of the Health Care Program. Management of Animal Care and Use Programs in Research, Education, and Testing.

[20] Laule G.E., Bloomsmith M.A., Schapiro S.J. (2003). The use of positive reinforcement training techniques to enhance the care, management, and welfare of primates in the laboratory. J. Appl. Anim. Welf. Sci..

[21] Bloomsmith M.A., Perlman J.E., Hutchinson E., Sharpless M., Weichbrod R.H., Norton J.N., Thompson G.A., Weichbrod R.H., Thompson G.A.H., Norton J.N. (2018). Behavioral Management Program to Promote Laboratory Animal Welfare. Management of Animal Care and Use Programs in Research, Education, and Testing.

[22] Schapiro S.J., Bloomsmith M.A., Laule G.E. (2003). Positive Reinforcement training as a technique to alter nonhuman primate behavior: Quantitative assessments of effectiveness. JAAWS.

[23] Schapiro S.J., Perlman J.E., Thiele E., Lambeth S. (2005). Training nonhuman primates to perform behaviors useful in biomedical research. Lab. Anim..

[24] Gillis T.E., Janes A.C., Kaufman M.J. (2012). Positive reinforcement training in squirrel monkeys using clicker training. Am. J. Primatol..

[25] Moseley J., Davis J. (1989). Psychological enrichment techniques and new world monkey restraint device reduce colony management time. Lab. Anim. Sci..

[26] Finnie K.R., Jones C.P., Dupont W.D., Salleng K.J., Shuster K.A. (2020). A comparison of the efficacy and cardiopulmonary effects of 3 different sedation protocols in Otolemur garnettii. J. Am. Assoc. Lab. Anim. Sci..

[27] Altmann J. (1974). Observational study of behavior: Sampling methods. Behaviour.

[28] McMillan J.L., Perlman J.E., Galvan A., Wichmann T., Bloomsmith M.A. (2014). Refining the pole-and-collar method of restraint: Emphasizing the use of positive training techniques with rhesus macaques (*Macaca mulatta*). J. Am. Assoc. Lab. Anim. Sci..

[29] Gilbert M.H., Baker K.C. (2011). Social buffering in adult male rhesus macaques (*Macaca mulatta*): Effects of stressful events in single versus pair housing. J. Med. Primatol..

[30] Jackson M.N., Truelove M.A., Williams K., Chen J., Moore R.H., Wood J.S., Cohen J.K., Bloomsmith M.A. (2023). Effects of pair housing on behavior, cortisol, and clinical outcomes during quarantine-like procedures for rhesus macaques (*Macaca mulatta*). J. Med. Primatol..

[31] Reinhardt V., Cowley D., Scheffler J., Vertein R., Wegner F. (1990). Cortisol response of female rhesus monkeys to venipuncture in homecage versus venipuncture in restraint apparatus. J. Med. Primatol..

[32] Reinhardt V., Cowley D. (1992). In-homecage blood collection from conscious stump tailed macaques. Anim. Welf..

[33] Reinhardt V., Liss C., Stevens C. (1995). Restraint methods of laboratory non-human primates: A critical review. Anim. Welf..

[34] Reinhardt V. (1997). Training nonhuman primates to cooperate during handling procedures: A review. Anim. Technol..

[35] Veeder C.L., Bloomsmith M.A., McMillian J.L., Perlman J.E., Martin A.L. (2009). Positive reinforcement training to enhance the voluntary movement of group-housed sooty mangabeys (*Cercocebus atys atys*). J. Am. Assoc. Lab. Anim. Sci..

[36] Rogge J., Sherenco K., Malling R., Thiele E., Lambeth S., Schapiro S., Williams L. (2013). Comparison of positive reinforcement training techniques in owl and squirrel monkeys: Time required to train to reliability. J. Appl. Anim. Welf. Sci..

